# First person – Nicoleta Baxan

**DOI:** 10.1242/dmm.041681

**Published:** 2019-08-16

## Abstract

First Person is a series of interviews with the first authors of a selection of papers published in Disease Models & Mechanisms (DMM), helping early-career researchers promote themselves alongside their papers. Nicoleta Baxan is first author on ‘Characterization of acute TLR-7 agonist-induced hemorrhagic myocarditis in mice by multi-parametric quantitative cardiac magnetic resonance imaging’, published in DMM. Nicoleta conducted the research described in this article while in Dr Susanne Sattler's lab at Imperial College London, UK. She is now an MR Physics Research Associate in the lab of Prof. Lan Zhao at Biological Imaging Centre, Imperial College London, UK, investigating the development of novel multi-parametric cardiac magnetic resonance (CMR) tissue-mapping techniques to make progress in the diagnosis and treatment of heart diseases.


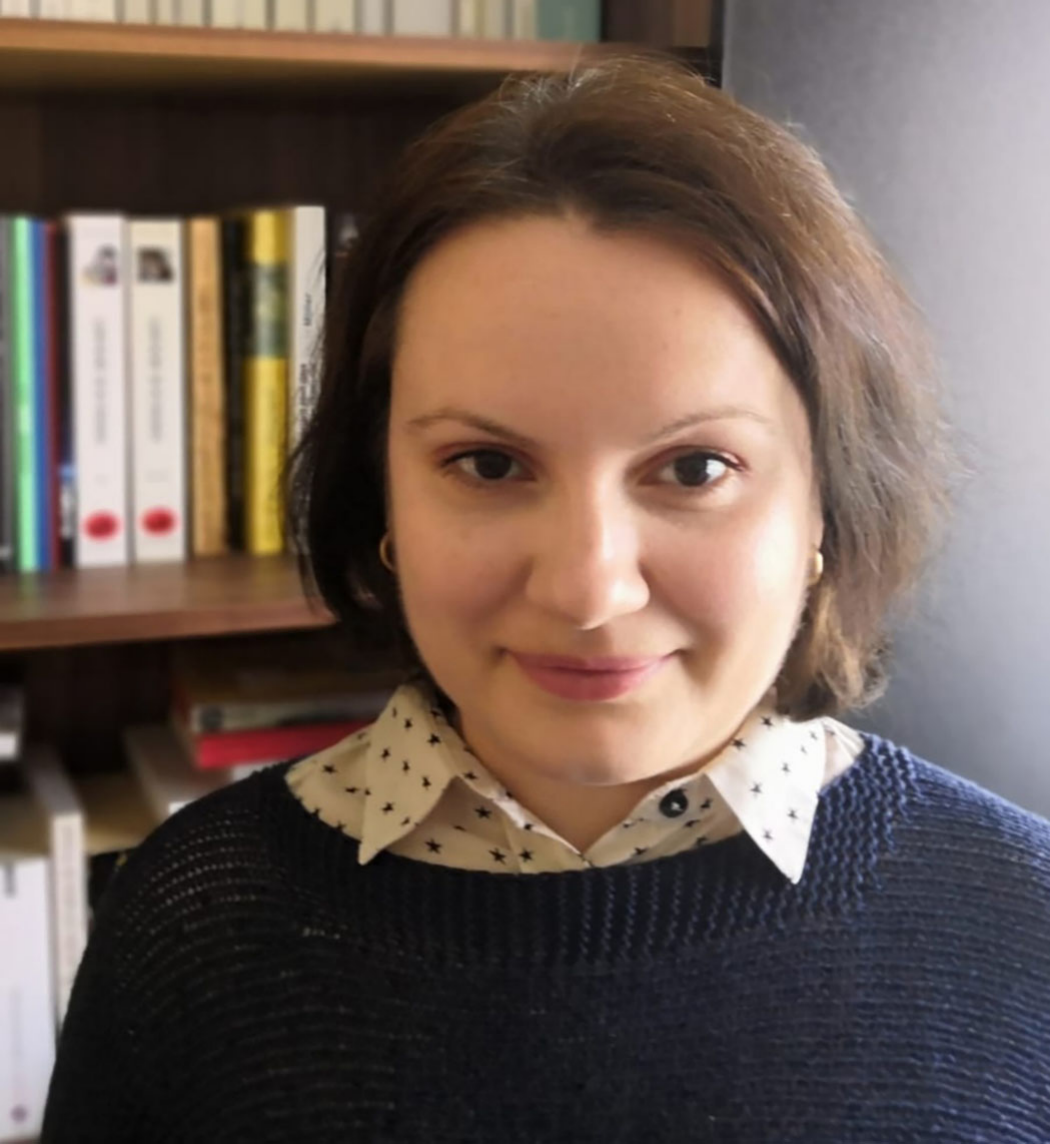


**Nicoleta Baxan**

**How would you explain the main findings of your paper to non-scientific family and friends?**

Immune-mediated damage to the heart may occur as the result of a wide variety of underlying conditions, including infectious disease, exposure to toxins, chemotherapeutic agents, and systemic inflammation due to autoimmune disease. CMR mapping provides unique information of tissue damage in inflammatory disease by tracking subtle changes in myocardium, such as specific disease pathways related to intracellular disturbances of cardiomyocytes (iron deposition); extracellular disturbances in the myocardial interstitium (fibrosis from accumulation of collagen); or both (myocardial edema and cellular infiltrate with increased intracellular and/or extracellular water). In this study, we identified a significant amount of interstitial iron in the hearts of Resiquimod-treated mice, which is most likely the result of hemorrhage, erythrocyte cell death and hemoglobin degradation. We show that chronic anti-heart autoimmunity in the Resiquimod model of systemic lupus erythematosus (SLE) follows acute thrombocytopenia and hemorrhagic myocarditis, and provide a thorough comparison of *in vivo* CMR parameter measurements with the underlying heart histopathology.

**What are the potential implications of these results for your field of research?**

Acute myocarditis may trigger an autoimmune reaction against the heart, and it is feasible that severity of hemorrhaging, acute tissue damage and subsequent autoimmunity are correlated. The ability to non-invasively discriminate different processes of tissue damage by using specific magnetic resonance imaging (MRI) metrics based on changes in myocardial parameters T_1_, T_2_, T_2_* will allow insight into the natural history of disease and improve our understanding of the potentially subclinical course of cardiac involvement in systemic inflammation.

“Studying genetically diverse mouse panels instead of a single inbred mouse line may reveal a range of susceptibilities.”

**What are the main advantages and drawbacks of the model system you have used as it relates to the disease you are investigating?**

Myocardial hemorrhage is not commonly reported in patients with myocarditis. Underlying mechanisms leading to hemorrhage in some myocarditis patients but not in others and implications on survival and subsequent development of inflammatory cardiomyopathies are far from understood. The specific phenotype of hemorrhagic myocarditis has only been studied in Resiquimod-induced systemic inflammation in *CFN* mice. Considering that myocardial hemorrhaging may be more common than currently appreciated in systemic inflammation of both infectious and autoimmune origin, as well as directly heart-targeted infections, these processes need to be characterized in the corresponding mouse models. Considering that only a small proportion of human patients develop clinically detected hemorrhagic myocarditis, there is likely to be a genetic component to disease susceptibility. Studying genetically diverse mouse panels instead of a single inbred mouse line may reveal a range of susceptibilities.

In acute stage, the mouse model used in this study closely mimics physiological responses to a viral infection due to the stimulation of the TLR-7 pathway. TLR-7 is a pattern-recognition receptor involved in recognition of single-stranded RNA of viral origin, and is thus crucial in host defense against viral infections. Artificial over-activation of this virus defense system may cause the same phenomena as seen in severe complications of viral infection (thrombocytopenia with local hemorrhages at sites of inflammation).

**Figure:**
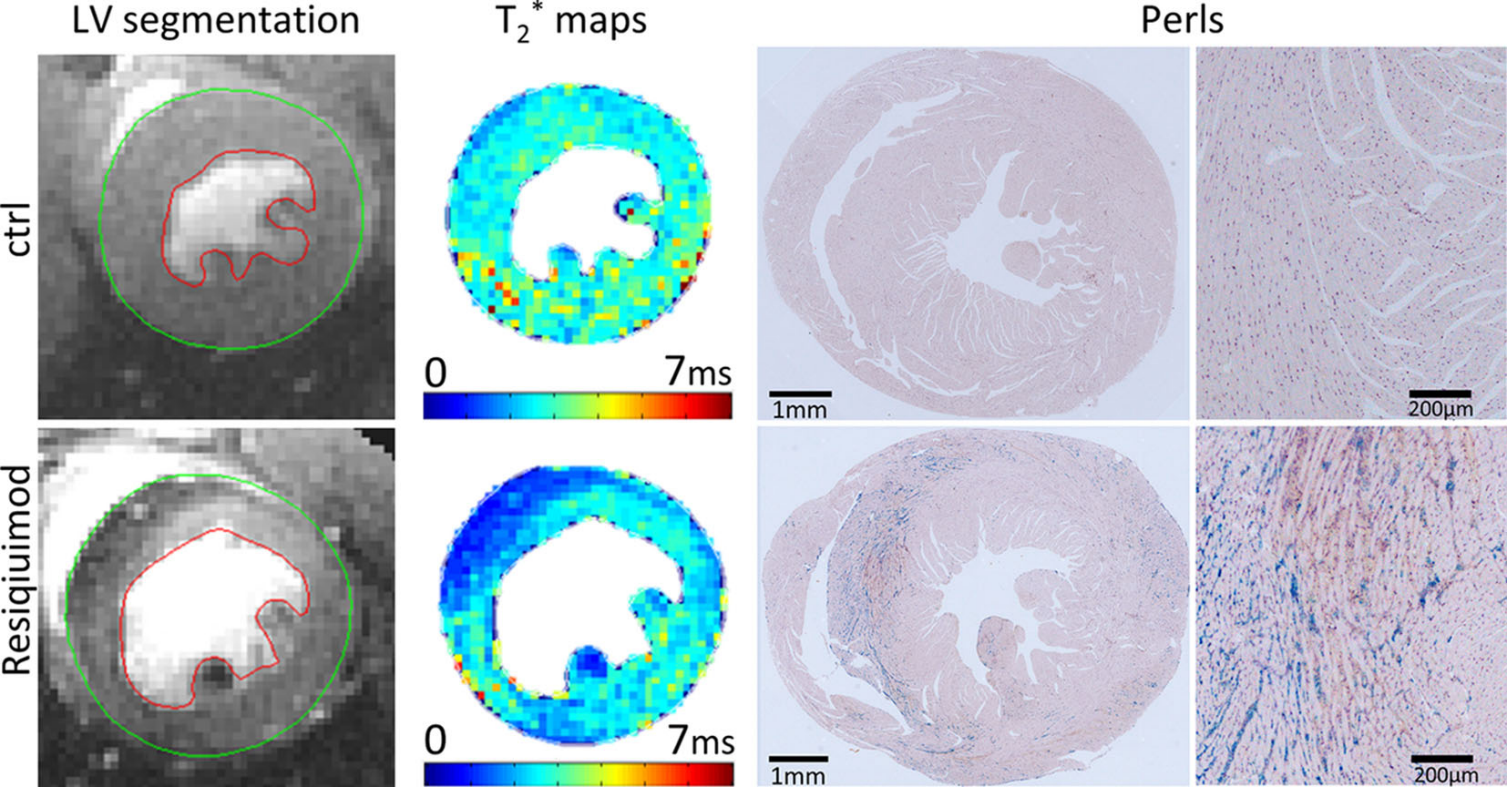
T_2_* mapping reveals severe cardiac iron deposition in Resiquimod-treated mice. Representative T_2_* maps of mid-section left ventricle with corresponding Perls Prussian Blue-stained paraffin-embedded heart sections of Resiquimod-treated and control mice showing significant iron deposition. High-magnification images of tissue areas with iron deposition (Perls Prussian Blue) are illustrated to the right.

**What has surprised you the most while conducting your research?**

I am constantly amazed by the power of multidisciplinary research, and this project is a true example of it. I am an MR physicist by training, and the collaborative nature of this project gave me the opportunity to work with amazing and highly skilled researchers coming from different disciplines (biology, clinical sciences, pharmacology, etc.). Bringing together multiple disciplines brought this paper to light (and most of my research so far), and this almost certainly wouldn't have been done by focusing on a single area.

“I am constantly amazed by the power of multidisciplinary research, and this project is a true example of it.”

**Describe what you think is the most significant challenge impacting your research at this time and how will this be addressed over the next 10 years?**

The technical challenges of acquiring CMR data synchronized with the cardiac motion combined with the relatively poor pathological specificity of CMR may affect the scan time, the analysis and interpretation of data. New emerging techniques based on artificial intelligence and machine learning approaches are bringing exciting possibilities of faster data acquisition and automated AI-driven quantitative analysis, eliminating many tedious, manual tasks. This will provide enormous opportunities to homogenize data acquisition and computation to better characterize disease by imaging.

“I believe that giving full confidence, responsibility and credit to early-career scientists to conduct their research independently is crucial for their future career.”

**What changes do you think could improve the professional lives of early-career scientists?**

I am very lucky to have a very supportive PI, giving me the chance to be autonomous in my work and in my projects. I am constantly learning and improving my skills of setting new collaborative projects, presenting my findings and networking. I believe that giving full confidence, responsibility and credit to early-career scientists to conduct their research independently is crucial for their future career.
